# *Molluscum pendulum* géant de la vulve chez une multipare

**DOI:** 10.11604/pamj.2021.39.113.29582

**Published:** 2021-06-08

**Authors:** Mamadou Gassama, Madou Sissoko

**Affiliations:** 1 Centre Hospitalier Universitaire de Dermatologie de Bamako, Bamako, Mali,; 2 Faculté de Médecine et d´Odontostomatologie de Bamako, Bamako, Mali

**Keywords:** *Molluscum pendulum*, géant, chirurgie, Molluscum pendulum, giant, surgery

## Abstract

Giant molluscum pendulums of the vulva are benign tumors, usually small in size. Larger lesions are rare and probably due to proliferation of mesenchymal cells within the hormone-sensitive subepithelial stromal layer of the lower genital tract. The size of the lesion represents an important functional and aesthetic prejudice. This rare discrete swelling deserves to be known because of its functional impact and/or uncertain evolution. We report a case of a 38-years-old female patient, multiparous G4P4V4 whose last child is 1-year-old, she consulted on 05th December 2018 for a large evolving mass in the left inguinal fold for 11 years. The interrogation did not find any particular medical-surgical history. Physical examination revealed a 20/10 cm mass at the base of the left labia majora, soft in consistency, painless, mobile in relation to the deep plane, and dotted with a hypochromic cicatricial macule (A and B). Endoanal examination found non reducible hemorrhoidal packets (B). The hypothesis of giant molluscum pendulum was evoked. The hypothesis of giant molluscum pendulum was evoked. The preoperative blood test showed a positive blood group B, HIV serology was negative, the hemoglobin level was 13.2 g/l, the feasible hemostasis test (TS-TC) was normal. We performed surgical excision of the tumor under general anesthesia. The specimen weighed 2500g (C). Histology showed edematous, myxoid, and richly vascularized connective tissue without cytonuclear atypia (D). We referred the patient to hepato-gastroenterology for management of the hemorrhoid.

## Image en médecine

*Molluscum pendulum* géant de la vulve sont des tumeurs bénignes, habituellement de petites tailles. Les lésions plus grandes sont rares et probablement attribuables à une prolifération de cellules mésenchymateuses au sein de la couche stromale sous-épithéliale sensible aux hormones du bas appareil génital. La taille de la lésion représente un préjudice fonctionnel et esthétique important. Cette tuméfaction rare discrète mérite d´être connue en raison de son retentissement fonctionnel et/ou de son évolution incertaine. Nous rapportons un cas d´une patiente de 38 ans, multipare G4P4V4 dont le dernier enfant a 1 an, elle a consulté le 05 décembre 2018 pour une grosse masse évolutive du pli inguinal gauche depuis 11 ans. L´interrogatoire n´a retrouvé aucun antécédent médico- chirurgical particulier. L’examen physique a retrouvé une masse de 20/10cm à la base de la grande lèvre gauche, de consistance molle, indolore, mobile par rapport au plan profond, et parsemée d’une macule hypo chromique cicatricielle (A et B). L'examen endo-anal a retrouvé des paquets hémorroïdaires non réductibles (B). L´hypothèse de *molluscum pendulum* géant a été évoquée. Le bilan sanguin pré opératoire a objectivé un groupe sanguin B rhésus positif, la sérologie HIV était négative, le taux d´hémoglobine: 13,2g/l, le bilan d'hémostase faisable (TS-TC) était normal. Nous avons effectué une exérèse chirurgicale de la tumeur sous anesthésie générale. La pièce pesait 2500g (C). L´histologie a montré un tissu conjonctif œdémateux, myxoïde et richement vascularisé sans atypie cytonucléaire (D). Nous avons envoyé la patiente en hépato-gastro-entérologie pour la prise en charge de l´hémorroïde.

**Figure 1:**
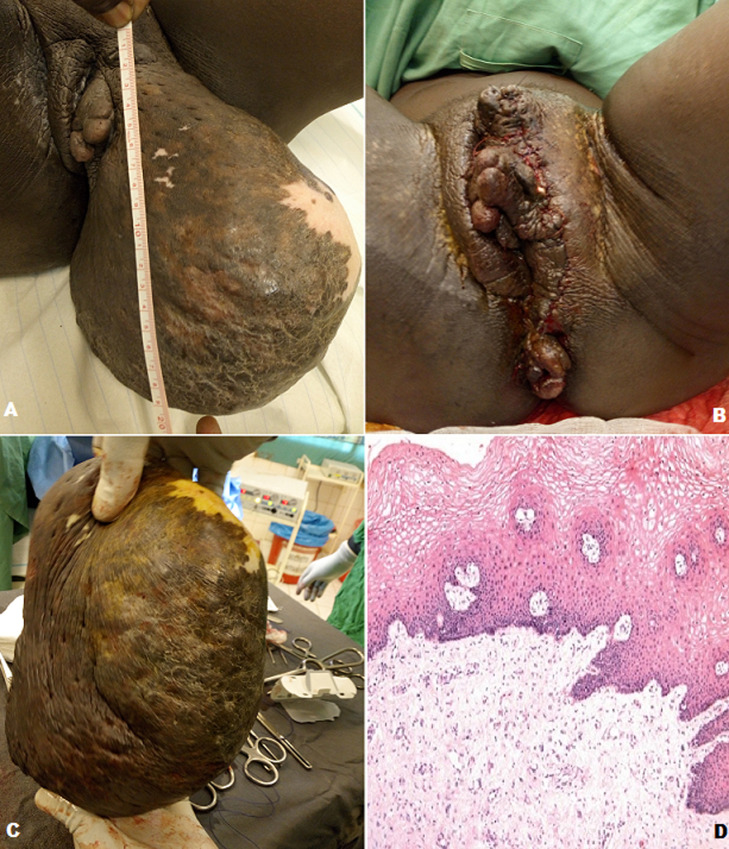
*molluscum pendulum* (A) avant chirurgie; (B) après chirurgie; (C) pièce opératoire; (D) aspect microscopique d´un tissu conjonctif œdémateux, myxoïde et richement vascularisé sans atypie cytonucléaire

